# Antimicrobial Susceptibility of Persister Biofilm Cells of *Bacillus cereus* and *Pseudomonas fluorescens*

**DOI:** 10.3390/microorganisms10010160

**Published:** 2022-01-13

**Authors:** Susana Fernandes, Inês B. Gomes, Sérgio F. Sousa, Manuel Simões

**Affiliations:** 1 LEPABE—Laboratory for Process Engineering, Environment, Biotechnology and Energy, Faculty of Engineering, University of Porto, Rua Dr. Roberto Frias, 4200-465 Porto, Portugal; sfernandes@fe.up.pt (S.F.); ibgomes@fe.up.pt (I.B.G.); 2 UCIBIO/REQUIMTE, BioSIM, Departamento de Biomedicina, Faculdade de Medicina da Universidade do Porto, Alameda Prof. Hernâni Monteiro, 4200-319 Porto, Portugal; sergiofsousa@med.up.pt

**Keywords:** benzalkonium chloride, biofilm control, disinfection, glycolic acid, glyoxal, peracetic acid, persisters

## Abstract

The present study evaluates the antimicrobial susceptibility of persister cells of *Bacillus cereus* and *Pseudomonas fluorescens* after their regrowth in suspension and as biofilms. Two conventional (benzalkonium chloride—BAC and peracetic acid—PAA) and two emerging biocides (glycolic acid—GA and glyoxal—GO) were selected for this study. Persister cells resulted from biofilms subjected to a critical treatment using the selected biocides. All biocide treatments developed *B. cereus* persister cells, except PAA that effectively reduced the levels of vegetative cells and endospores. *P. fluorescens* persister cells comprise viable and viable but non-culturable cells. Afterwards, persister cells were regrown in suspension and in biofilms and were subjected to a second biocide treatment. In general, planktonic cultures of regrown persister cells in suspension lost their antimicrobial tolerance, for both bacteria. Regrown biofilms of persister cells had antimicrobial susceptibility close to those regrown biofilms of biocide-untreated cells, except for regrown biofilms of persister *P. fluorescens* after BAC treatment, which demonstrated increased antimicrobial tolerance. The most active biocide against persister cells was PAA, which did not promote changes in susceptibility after their regrowth. In conclusion, persister cells are ubiquitous within biofilms and survive after critical biocide treatment. The descendant planktonic and biofilms populations showed similar properties as the original ones.

## 1. Introduction

Biofilms are characterized by a highly dense microbial community adhered to surfaces. These microorganisms are typically intricated in a self-produced matrix of extracellular polymeric substances (EPSs) [1,2]. Bacteria within biofilms are less susceptible to the antimicrobial activity of biocides than their planktonic counterparts [3]. Hence, sessile bacteria will survive and persist on surfaces even after cleaning and disinfection [4,5]. The protection conferred by biofilm structures is complex and involves the combination of multiple factors [6]. Firstly, the biofilm structure with an EPS matrix protects cells from antimicrobial activity by chemical-diffusion limitations [7]. For instance, the biocide level that reaches the inner biofilm layers is significantly reduced, resulting in distinct environments, from lethal to sub-lethal conditions. Another aspect is related to the development of an active (energy-dependent) stress response by biofilm cells (i.e., DNA damage repair, catalase induction, modification of cell membrane, and over-expression of efflux pumps) [8,9]. Finally, changes in susceptibility could occur due to the presence of persister and/or resistant cells [10,11].

Persister cells are characterized by a transient antimicrobial-tolerant phenotype [12]. Kaldalu et al. [13] defined persister cells as “individual bacteria that survive antibiotic treatment, which otherwise kills the large majority of their kin population”, corresponding to a “minor subpopulation of bacteria that are transiently tolerant to the lethal activity of antimicrobials”. In contrast, resistant cells are bacteria able to survive treatment with increased concentrations of bactericidal antibiotics through active adaptations, such as mutations, which are genetically transferred to new cells [13]. Within biofilms, persister cells appear stochastically rather than being produced in response to antimicrobial activity [14]. The specificity in the mode of action of antibiotics can be affected by the reduced metabolic activity of a subset of the bacterial population without undergoing mutations—known as dormant cells and typically named persister cells [15]. However, the antimicrobial activity of biocides is multi-target and usually independent from the bacterial metabolic state. Biocides may promote the death of dormant bacterial cells [16,17]. Thus, persister cells from biocide treatment may not include that dormant population, critical for antibiotic therapy. As a minor fraction of the biofilm population after biocide treatment, persister cells remain viable as surviving cells and resume growth, giving rise to a new biofilm mainly composed of susceptible cells (with similar susceptibility to the ancient population) [18]. Detached persister biofilm cells quickly convert into susceptible planktonic counterparts. Behnke et al. [19] reported a distinct and transitional phenotype between biofilm, detached, and planktonic cells, with different antimicrobial susceptibilities to chlorine.

The understanding of biofilm tolerance and the development of persister cells are huge challenges for surface disinfection. The occurrence of persister cells is predominantly mentioned in the medical context, focusing on the survival of pathogenic bacteria after antibiotic treatment [20,21]. Moreover, the development of persister cells during disinfection processes is still underexplored. Only a few studies have demonstrated the presence of persister cells within biofilms after disinfection. For example, Simões et al. [10] and Ng et al. [22] verified the presence of persister cells within biofilm after treatment using *ortho*-phthalaldehyde and monochloramine, respectively. A mathematical simulation of the effectiveness of biofilm treatment by periodic and continuous dosing protocols of biocides was applied by Cogan et al. [23], considering both susceptible and persister cells. The present study aims to evaluate the presence of persister cells in biofilms exposed to extreme treatments with conventional (benzalkonium chloride—BAC and peracetic acid—PAA) and underexplored biocides (glycolic acid—GA and glyoxal—GO). Additionally, the antimicrobial susceptibility of persister cells was evaluated. In order to understand the impact of persister cells on the contamination of products and on the development of new biofilms, the regrowth of persister cells was performed in a planktonic state and as a biofilm. Then, a second biocide treatment was performed against both types of cultures to evaluate if regrown cultures from persister cells present an altered susceptibility profile.

## 2. Materials and Methods

### 2.1. Bacterial Strains and Culture Conditions

The bacterial strains selected as representative of food spoilage microorganisms were *Bacillus cereus* strain that was isolated from a disinfectant solution and identified by 16 S rRNA gene sequencing [24] and *Pseudomonas fluorescens* ATCC 13525T. Overnight cultures of both bacteria were grown using a sterile synthetic nutrient medium (5 g/L glucose, 2.5 g/L peptone and 1.25 g/L yeast extract, in 0.2 M phosphate buffer, pH 7) at 30 ± 3 °C and under agitation (120 rpm). All components were purchased from Merck (Darmstadt, Germany). The absence of endospores in the overnight grown culture of *B. cereus* was confirmed by CFU counting after heat treatment (80 °C, 5 min), according to Simões, et al. [25]. Overnight grown cultures (called regular planktonic cultures) were centrifuged for 10 min (3772× *g*) and washed with phosphate buffer saline (PBS: 8 g/L NaCl—VWR, Leuven, Belgium, 0.2 g/L KCl—VWR, Leuven, Belgium, 1.44 g/L Na_2_HPO_4_ and 0.24 g/L KH_2_PO—Chem-Lab NV, Zedelgem, Belgium, pH 7.4). The cell pellets were resuspended in tryptic soy broth (TSB—Merck, Darmstadt, Germany) or PBS, and adjusted to 8-log colony-forming units per mL (CFU/mL).

### 2.2. Biocides

The selected conventional biocides were benzalkonium chloride (BAC, Sigma-Aldrich, Søborg, Denmark) and peracetic acid 38–40% (*w/v*) (PAA; Merck, Darmstadt, Germany); and the emerging biocides were glycolic acid 99% (*w/w*) (GA, Sigma-Aldrich, Burlington, MA, USA) and glyoxal 40% (*w/v*) (GO, Sigma-Aldrich, Darmstadt, Germany). Biocidal solutions were freshly prepared in sterile distilled water (DW) at the selected concentrations (Table 1 and Table 2).

### 2.3. Experimental Setup

**Figure 1:**
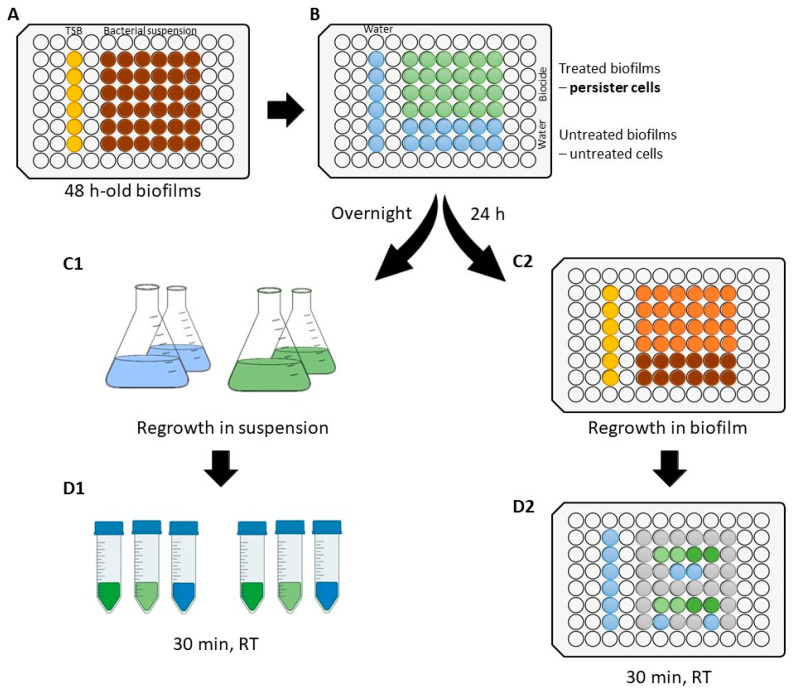
Experimental setup to achieve the antimicrobial activity of each biocide against regrown persister cells. (**A**)—48-h-old biofilm formation; (**B**)—optimized biocide treatment for the development of persister cells; (**C1**)—cultures of regrown cells in suspension (untreated and persister cells); (**D1**)—antimicrobial activity against regrown cells in suspension (30 min of exposure under room temperature, RT); (**C2**)—regrown biofilms of untreated and persister cells; (**D2**)—antimicrobial activity against regrown biofilm cells (30 min, RT).

Figure 1 represents the experimental setup followed to develop persister cells and to evaluate their antimicrobial susceptibility. Initially, 48-h-old biofilms of *B. cereus* and *P. fluorescens* were produced (Figure 1A). Persister cells were obtained after a critical biocide treatment (Figure 1B), as described in Section 2.4. Then, that population was regrown in suspension (Figure 1C1) and biofilms (Figure 1C2), followed by a second biocide treatment to assess their antimicrobial susceptibility (Section 2.5). Firstly, the antimicrobial susceptibility of regrown cells in suspension (of untreated cells and persister cells) was compared to that of the regular planktonic population (from overnight grown cultures—Section 2.1) (Figure 1D1; see Section 2.5.1). Finally, the antimicrobial susceptibility of regrown biofilms of persister cells was compared to that of regrown biofilms of biocide-untreated cells (Figure 1D2; see Section 2.5.2). For that, all cultures were exposed to two distinct concentrations of each biocide selected (exposed cells).

### 2.4. Development of Persister Cells after Biocide Treatment

Biofilms in 96-well polystyrene microtiter plates were developed according to a method adapted from Stepanovic, et al. [26] (Figure 1A). For each bacterial strain, each well was filled with 200 µL of bacterial suspension in TSB. Growth media without cells was used as negative control. The plates were incubated aerobically for 48 h at 30 ± 3 °C, under static conditions. Then, the culture medium was carefully discarded and replaced by a fresh one, and the plates were incubated for more 24 h. Afterwards, each well was empty and once washed with 250 µL of DW to remove non-adhered and weakly adhered cells. The isolation of persister cells (as surviving cells) was performed according to the method applied by Pan, et al. [27]. Briefly, biocide treatment was performed at room temperature for a defined exposure time by adding 200 µL of biocidal solution per well. Biocide exposure concentration and time (Table 1) were optimized to obtain the maximum antibiofilm activity. For instance, considering 30 min of exposure time, the complete antibiofilm activity was reached by PAA at 10,000 µg/mL against *B. cereus* biofilms, and by PAA at 20,000 µg/mL and GA at 40,000 µg/mL against *P. fluorescens* biofilms. For the other conditions tested, as increasing biocide concentration did not cause a significant reduction of log CFU/cm^2^ (Figure S1), an additional increase of exposure time was carried out from 30 min to 4 h (Figure S2). In general, no additional killing was observed from increasing exposure time, except for BAC at 1000 µg/mL against *P. fluorescens*. The concentrations and exposure times tested are listed in Table 1. After biocide treatment, each well was empty and neutralized for 15 min with 40 µL of DW and 160 µL of universal neutralizer (30 g/L polysorbate 80 (VWR Chemicals, Le Havre, France), 30 g/L saponin (VWR Chemicals, Leuven, Belgium), 1 g/L L-histidine (Merck, Tokyo, Japan), 3 g/L lecithin (Alfa Aesar, Karlsruhe, Germany), 5 g/L sodium thiosulphate (Labkem, Barcelona, Spain) in 0.0025 M phosphate buffer [28] (Figure 1B). Untreated biofilm cells were obtained by exposing biofilms to DW instead of biocides. Treated (persister cells) and untreated biofilms (untreated cells) were analyzed in terms of cell culturability.

For that, biofilms were scraped three times from the surfaces for 1 min using a pipette tip [29,30], resuspended in 1 mL of sterile saline solution (0.85% *w/v* NaCl) and vigorously vortexed for 30 s [31]. The cell culturability was determined by CFU counts onto TSA plates after incubation at 30 ± 3 °C for 24 h. The results are represented as log CFU/cm^2^. The limit of detection was 1.5-log CFU/cm^2^. *B. cereus* was analyzed in terms of endospores by CFU counting [25] to differentiate surviving cells between endospores and persister cells. For *P. fluorescens*, when no culturable cells were detected; persister cells were also quantified in terms of viable cells using a Live/Dead BacLightTM kit (Invitrogen/Molecular Probes), as described by Borges et al. [32].

### 2.5. Antimicrobial Activity against Regrown Cultures

The impact of persister cells on the contamination of products and biofilm resilience need to be explored; therefore, the regrowth of persister cells was performed in suspension (Section 2.5.1) and as biofilms (Section 2.5.2). Then, a second biocide treatment was performed against both types of cultures to evaluate if regrown cultures from persister cells present an altered susceptibility profile in comparison to the original ones.

#### 2.5.1. Susceptibility of Regrown Cultures in Suspension

After biocide treatment and neutralization, treated biofilms (persister cells, which include endospores in the case of *B. cereus*) and untreated biofilms were detached from the surfaces (as in Section 2.4) and used to inoculate fresh growth media. These populations were grown overnight using a sterile synthetic nutrient medium (100 and 50 mL for *B. cereus* and *P. fluorescens*, respectively) at 30 ± 3 °C under agitation (120 rpm). The absence of endospores in the overnight regrown cultures of *B. cereus* was confirmed [25]. The regular and regrown cultures were centrifuged, washed, and resuspended in PBS. Then, the cell suspensions were adjusted to approximately 8-log CFU/mL in PBS (Figure 1C1). Afterwards, 1 mL of cell suspension was mixed with 1 mL of DW and maintained in contact for 2 min. Then, 8 mL of biocidal solution was added and remained in contact for 30 min at room temperature (Figure 1D1). The tested biocide concentrations are described in Table 2. Control samples corresponded to replace biocidal solution by DW. After exposure, biocide neutralization was performed according to EN 1276 [28] through the dilution–neutralization method using a universal neutralizer. Then, the remaining cells were determined by CFU counting onto TSA plates after incubation at 30 ± 3 °C for 24 h. For cell recount, plates were incubated for a further 24 h. The detection limit was 2.7-log CFU/mL. The antimicrobial activity was quantified by log CFU/mL reduction as log(X/X_0_), in which X_0_ is the counts of CFU/mL for biocide-unexposed bacteria and X is the counts of CFU/mL of exposed bacteria. The susceptibility of regrown cultures in suspension was not assessed for all the conditions tested since the regrowth of persister cells of biofilms treated with bactericidal concentrations of GA and PAA had no culturable cells in the planktonic culture. Additionally, regrown persister *P. fluorescens* in suspension after GO treatment showed low overnight growth, without enough culturable cells for the following antimicrobial susceptibility assays.

#### 2.5.2. Susceptibility of Regrown Biofilms

After biocide treatment and neutralization, each well containing treated (persister cells, which include endospores in the case of *B. cereus*) and untreated biofilms was aseptically filled with 200 µL of TSB and incubated for 24 h at 30 °C, under static conditions. Afterwards, the antimicrobial susceptibility of regrown biofilms was assessed as described in Section 2.4 (Figure 1D2). Each population of regrown biofilms of persister cells after each biocide treatment were exposed to each biocide at two distinct concentrations (Table 2). The regrown biofilms of untreated cells were exposed to all the selected biocides. Positive controls were performed using DW instead of the biocidal solutions. The surviving cells (in %) were determined as X/X_0_ × 100, in which X_0_ is the counts of CFU/cm^2^ for the positive control and X is the counts of CFU/cm^2^ for exposed biofilms.

### 2.6. Statistical Analysis

Data were analyzed using GraphPad Prism 6.0 for Windows (GraphPad Software, La Jolla, CA, USA). The mean and standard deviations (SDs) within samples were calculated for all cases. Statistically significant differences were established for a probability level of 5% (*p* < 0.05). Two distinct samples were compared by the application of unpaired *t*-tests with Welch’s correction. Distinct samples and a control sample were compared by the application of Dunnett’s multiple comparisons test.

## 3. Results and Discussion

### 3.1. Persister Biofilm Cells after Biocide Treatment

Biocides act against multiple cellular targets and may promote the death of persister cells (in particular, dormant cells) since their antimicrobial activity is independent of the metabolic state [16,17]. However, several protective factors within the biofilm structure keep the persister cells away from the biocide action. For example, the physicochemical interactions between the biocide and EPS components can reduce the active concentration that reaches the inner cells [2,33]. Nevertheless, the regrowth of persister biofilm cells, even without the EPS matrix, after treatment with bactericidal concentrations has been also reported [10]. Other protective factors could be related to cell communication (quorum-sensing mechanisms) and mass transfer limitations [34]. Thus, the behavior and development of persister cells within biofilms after biocide treatment remains to be understood. In the current study, the presence of persister cells within biofilms of *B. cereus* and *P. fluorescens* was assessed after critical biocide treatments using BAC, GA, GO, and PAA. The antibiofilm activity of the selected biocides is reported elsewhere [35]. The authors demonstrated a similar mode of action between BAC/GO and PAA/GA. However, the effective concentrations of GA and GO were higher when compared to BAC and PAA.

**Figure 2:**
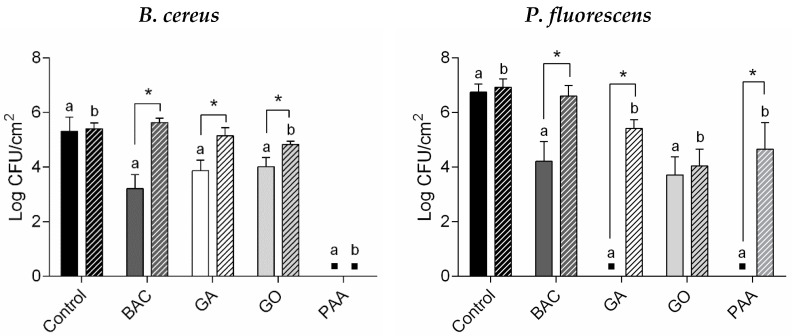
Persister cells of *B. cereus* and *P. fluorescens* after BAC, GA, GO, and PAA treatment (continuous bars) and regrown biofilms (dashed bars). Control corresponds to the untreated biofilms. Values are mean ± SDs of three independent assays with two replicates. ▪—No culturable persister/regrown cells were detected (<1.5-log CFU/cm^2^). *—Initial persister cells were statistically different from regrown biofilms of persister cells (unpaired *t*-test with Welch’s correction, *p* < 0.05); a—persister cells were statistically different from untreated cells (control) (Dunnett’s multiple comparisons test, *p* < 0.05); b—regrown biofilms of persister cells were statistically different from regrown biofilms of untreated cells (control) (Dunnett’s multiple comparisons test, *p* < 0.05).

After biocide treatment using optimized concentrations and exposure times (which ensured a complete or maximum antibiofilm activity—Table 1), persister cells, quantified as surviving cells, were present within the biofilms of both bacteria (Figure 2). Persister development was found to be biocide- and strain-dependent. The most active biocide against biofilm cells of both bacteria, including these persisters, was PAA. Against *B. cereus* biofilms, only the exposure to PAA did not allow the development of persister cells since this biocide affected both vegetative cells and endospores. In the case of *B. cereus*, it was also possible to detect endospores among the total CFU counts (Table S1). These endospores may also be included in the total number of persister cells presented in Figure 2. Despite the treatment applied, the endospores counts were approximately 3.2-log CFU/cm^2^, even for untreated biofilms (except for PAA, which caused the complete control of endospore formation). This means that the counted persister cells contain endospores (approximately 30% of the counted persister *B. cereus* were endospores). Against *P. fluorescens* biofilms, GA and PAA exposure allowed no culturable cells recovery (Figure 2); however, around 4.8-log viable cells/cm^2^ remained after treatment (6.5-log total cells/cm^2^; see Table S2). This suggests the presence of viable but non-culturable cells.

Persister cells of *B. cereus* and *P. fluorescens* biofilms were regrown in suspension and biofilms. All biofilms without detectable culturable persister cells (*B. cereus* treated with PAA and *P. fluorescens* treated with GA and PAA) did not grow overnight in suspension. Moreover, regrown persister *P. fluorescens* in suspension after GO treatment also had low overnight growth, which could be explained by the impact of GO on the microbial growth rate, as previously described by Fernandes et al. [36]. Regrown *B. cereus* persister cells in suspension had no detectable endospores. Regarding regrown biofilms, persister *B. cereus* was able to develop biofilms with similar cell densities of untreated cells (see Figure 2; *p* > 0.05). Only regrown biofilms of persister *B. cereus* after GO treatment showed lower cell density than regrown biofilms of untreated cells (*p* < 0.05). As the antimicrobial activity of PAA was effective against vegetative cells and endospores of *B. cereus* (Table S1), there was no regrowth of their persister cells. Although there was an undetectable presence of culturable bacteria, persister *P. fluorescens* after GA and PAA treatment ended up on regrown biofilms with lower cell culturability than the untreated counterparts (*p* < 0.05). This behavior was explained by the presence of viable but non-culturable cells (4.8-log viable cells/cm^2^) that can reseed a biofilm when favorable conditions are established [37]. However, at the same point, these persister cells were not able to regrow when freely suspended. The authors suggested a mechanism from the previous latent state that ended by inducing cell death, due to the lack of cell communication (QS), as a consequence of the spatial cell distance. As reviewed by Li and Zhao [38], QS is crucial in the regrowth of viable but non-culturable cells. So, QS inhibition is used to prevent their regrowth and promote cell death [38]. Controversially, another study demonstrated that persister *P. fluorescens* from a bactericidal exposure to *ortho*-phthaldehyde regrew in suspension after incubation for 24 h [10]. These differences could be explained by the mode of action of the biocides used, which might impact membrane permeabilization and cell growth differently [39].

### 3.2. Antimicrobial Susceptibility of Regrown Cells in Suspension

**Figure 3:**
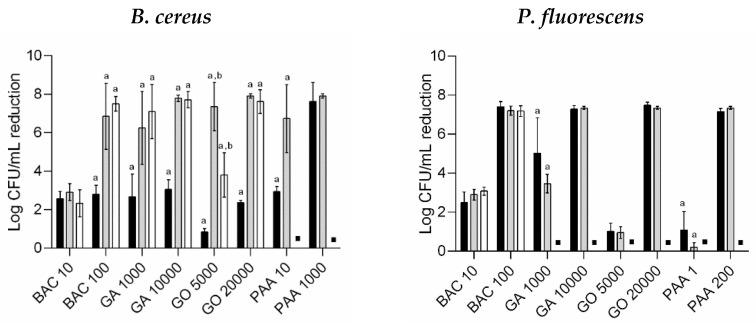
Antimicrobial activity of the selected biocides at different concentrations (in µg/mL) against regular planktonic culture (in black), regrown cultures of untreated cells (in grey) and persister cells (in white) of *B. cereus* and *P. fluorescens*. Values are mean ± SDs of three independent assays with two replicates. ▪—no detectable/significant cell growth after overnight incubation. a—regrown cultures were statistically different from regular planktonic cultures (Dunnett’s multiple comparisons test, *p* < 0.05); b—regrown cultures of untreated cells were statistically different from those from persister cells (unpaired *t*-test with Welch’s correction, *p* < 0.05).

The results from the antimicrobial activity of the selected biocides at different concentrations against suspension cultures of *B. cereus* and *P. fluorescens* (regular planktonic cultures, regrown cultures of untreated and persister cells) are presented in Figure 3. Independently from any previous biocide treatment, regrown *B. cereus* (from persister cells and endospores) in suspension had higher antimicrobial susceptibility than the regular counterparts (*p* < 0.05). Endospores from *B. cereus* biofilms will germinate and convert into vegetative cells when favorable environmental conditions are established [40]. The new vegetative bacteria are again active and susceptible to the antimicrobial activity of biocides [40]. No significant differences were found between the susceptibility of the regular planktonic cultures of *P. fluorescens* and regrown *P. fluorescens* in suspension (*p* > 0.05), which demonstrated the return to a susceptible planktonic state due to the loss of mechanisms of protection typical of biofilms [34]. The exception was regrown *P. fluorescens* exposed to 1000 µg/mL of GA and 1 µg/mL of PAA, which showed lower log CFU/mL reduction in comparison to the regular planktonic culture (*p* < 0.05). However, the susceptibility remained similar for the bactericidal concentrations of these biocides (10,000 and 200 µg/mL of GA and PAA, respectively) (*p* > 0.05). Regrown persister *P. fluorescens* after BAC treatment lost their tolerance and returned to their susceptible state as regular planktonic culture. The reversible switch between tolerant biofilm and susceptible planktonic counterpart has also been demonstrated using several antibiotics [41] and a biocide (*ortho*-phthalaldehyde) [10]. It was not possible to assess the susceptibility of regrown persister cells in suspension after GA, GO, and PAA treatment since no overnight growth was observed.

In general, the antimicrobial susceptibility of regrown cultures of untreated and persister cells was statistically similar (*p* > 0.05). The only exception was *B. cereus* exposed to GO at 5000 µg/mL, in which regrown persister cells (including cells from endospores) were less susceptible than untreated cells, but both remained more susceptible than the regular planktonic culture (*p* < 0.05). Thus, for both bacteria, biofilm detachment followed by biocide treatment is a promising strategy for biofilm control, i.e., a strategy based on cleaning; biofilm removal (wiping, scrubbing, flushing, heat shock treatment, and enzymes [42]), followed by disinfection, may be an effective approach to ensure microbial safety in surfaces contaminated by biofilms. Gomes et al. [43] achieved a synergistic behavior by increasing biofilm removal through the combination of quaternary ammonium compound (QAC) and mechanical treatment (shear stress from 0.7 to 17.7 Pa). After biofilm detachment, cells must encounter a bactericidal environment to prevent cell dissemination and biofilm formation.

### 3.3. Antimicrobial Susceptibility of Regrown Biofilms

**Figure 4:**
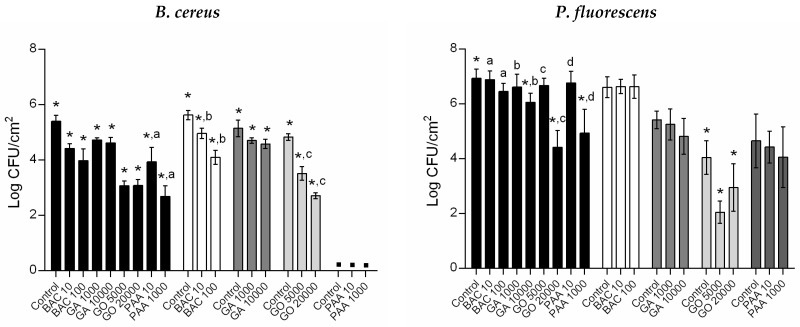
Log CFU/cm^2^ for exposed and unexposed (control) regrown biofilms of untreated and persister cells of *B. cereus* and *P. fluorescens*. Each selected biocide (BAC, GA, GO, and PAA) was tested at two distinct concentrations (in µg/mL). Values are mean ± SDs of three independent assays with two replicates. Legend: regrown biofilms of untreated cells (■); regrown biofilms of persister cells after BAC treatment (□), GA treatment (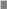), GO treatment (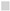), and PAA treatment (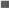). ▪—no detectable/significant regrown biofilms. *—Surviving cells from biocide exposure were significantly lower compared to unexposed regrown biofilms (control) for each population (Dunnett’s multiple comparisons test, *p* < 0.05); a, b, c, and d—Surviving cells were significantly different from distinct biocide concentration exposure (unpaired *t*-test with Welch’s correction, *p* < 0.05).

The antimicrobial susceptibility of regrown biofilms of untreated and persister cells is presented in Figure 4. All tested conditions caused a significant reduction in *B. cereus* biofilm culturability (*p* < 0.05). Considering regrown *P. fluorescens* biofilms of untreated cells, only the highest concentrations tested of GA, GO, and PAA caused a significant reduction in bacterial culturability (*p* < 0.05). Furthermore, regrown persister *P. fluorescens* biofilms had similar culturability as the unexposed counterparts (*p* > 0.05). The exception was the regrown biofilms of persister *P. fluorescens* following GO treatment, which significantly reduced bacterial culturability in comparison to the unexposed control (*p* < 0.05).

As regrown biofilms from untreated and persister cells showed significant differences in culturability, dependent on the primal biocide treatment and bacteria strain (Figure 2), the differences in the antimicrobial susceptibility between these populations were accomplished by determining the percentage of surviving cells (%; see Table 3). In general, all regrown biofilms from both bacteria had similar antimicrobial susceptibility for the conditions tested (*p* > 0.05), except the regrown biofilms of persister cells following GO treatment for both bacteria and BAC treatment for *P. fluorescens*. Regrown biofilms of persister cells following GO treatment revealed opposite effects, dependent on the bacteria. Thus, for 5000 µg/mL of GO, regrown *B. cereus* biofilms of persister cells were less susceptible (high % surviving cells), while those of *P. fluorescens* were more susceptible (less % surviving cells) than regrown biofilms of untreated cells (*p* < 0.05). Persister *P. fluorescens* did not significantly increase biofilm culturability after regrowth for 24 h (Figure 2). Thus, additional damages on these cells were induced in the following GO exposure. However, the highest GO concentration (20,000 µg/mL) tested caused similar effects against both bacteria (*p* > 0.05). In addition, regrown *P. fluorescens* biofilms of persister cells following BAC treatment were less susceptible (high % surviving cells) to the antimicrobial activity of the biocide at 100 µg/mL (*p* < 0.05) than its untreated counterparts, demonstrating a potential to develop tolerance. This increase in tolerance could be a result of phenotypic changes that were lost when freely dispersed since the planktonic counterparts retained the same susceptibility as the regular planktonic population. The tolerance development from the persistence against BAC has been the subject of other studies, as reviewed by Kampf [44]. Other authors also demonstrated the presence of phenotypically tolerant persister subpopulation to the antimicrobial activity of BAC and consequent cross-resistance to antibiotic action [45,46]. For example, Nordholt et al. [45] verified an increase of BAC tolerance by *Escherichia coli* through reduction of cell surface charge and mutations in an enzyme for lipid A biosynthesis. These features were involved in selective advantages in the presence of antibiotics [45].

## 4. Conclusions

Persister cells developed after critical biocide treatment are comprised of vegetative cells, endospores, and viable but non-culturable cells. The type and distribution of persister cells are dependent on the biocide treatment and bacteria. This study demonstrates that a critical biocide treatment does not ensure total antimicrobial activity and that persister cells (including endospores in the case of *B. cereus)* will be resilient on the surfaces. When persister cells switch to the planktonic state after biofilm detachment and regrow in suspension, the increased tolerance verified in biofilm is lost, returning to the susceptible state. In general, after biocide treatment, persister cells were able to develop a new biofilm with similar susceptibility as the original one. The exception verified was persister *P. fluorescens* after BAC treatment, which regrew into a less susceptible biofilm. Additionally, GO treatment also induced a slight decrease in the susceptibility of regrown *B. cereus* biofilms. In general, persister cells after GA and PAA treatment did not change their antimicrobial activity after regrowth in suspension and in biofilm. As PAA causes high antimicrobial activity with total *B. cereus* biofilm control, it can be considered the most active biocide against persister cells. Overall, the present results support that a potential setup for effective biofilm control considers surface cleaning (biofilm detachment) and disinfection. Moreover, the establishment of a regular disinfection schedule may be crucial to limiting the microbial load on the target surface to a safe level. The present results highlight that a regular biocide application should not induce changes in antimicrobial susceptibility since, for the biocides studied, the persister cells in planktonic and biofilm states had similar susceptibility as the preceding cells.

## Figures and Tables

**Table 1:** Experimental conditions (biocide concentration and exposure time) for the development of persister cells in *B. cereus* and *P. fluorescens* biofilms.

	*B. cereus*		*P. fluorescens*	
	Concentration (µg/mL)	Exposure Time (h)	Concentration (µg/mL)	Exposure Time (h)
BAC	500	0.5	1000	4
GA	1000	0.5	40,000	0.5
GO	20,000	0.5	20,000	0.5
PAA	10,000	0.5	20,000	0.5

**Table 2:** Concentrations (in µg/mL) of the selected biocides used against regrown persister cells in suspension and biofilm.

	*B. cereus*		*P. fluorescens*	
	Suspension	Biofilm	Suspension	Biofilm
BAC	10, 100		10, 100	
GA	1000, 10,000		1000, 10,000	
GO	5000, 20,000		5000, 20,000	
PAA	10, 1000		1, 200	10, 1000

**Table 3:** Antimicrobial activity in terms of surviving cells (%) of selected biocides at different concentrations (in µg/mL) against regrown biofilms of untreated and persister cells of *B. cereus* and *P. fluorescens*. Values are mean ± SDs of three independent assays with two replicates.

		*B. cereus*		*P. fluorescens*	
		Untreated	Persister	Untreated	Persister
BAC	10	12 ± 7	28 ± 10	72 ± 23	78 ± 25
	100	4 ± 3	4 ± 3	28 ± 10 *	77 ± 22 *
GA	1000	20 ± 5	37 ± 23	52 ± 33	64 ± 29
	10,000	16 ± 4	34 ± 26	25 ± 21	40 ± 27
GO	5000	0.5 ± 0.3 *	6 ± 3 *	44 ± 24 *	2 ± 3 *
	20,000	0.6 ± 0.3	0.8 ± 0.3	0.5 ± 0.7	9 ± 9
PAA	10	5 ± 4	--	58 ± 30	35 ± 21
	1000	0.2 ± 0.1	--	2 ± 4	23 ± 24

* Surviving cells (%) of regrown biofilms of persister cells were statistically different from regrown biofilms of untreated cells (unpaired *t*-test with Welch’s correction, *p* < 0.05).

## Data Availability

Not applicable.

## Supplementary Materials

The following are available online at https://www.mdpi.com/article/10.3390/microorganisms10010160/s1, Figure S1: Log CFU/cm^2^ of 48-h-old *B. cereus* (in black) and *P. fluorescens* biofilms (in grey) treated with selected biocides (BAC, GA, GO, and PAA) at different concentrations (in µg/mL) for 30 min. Values are means ± SDs of three independent experiments, Figure S2: Antimicrobial activity of BAC, GA, and GO at high concentrations (in µg/mL) for 30 min (full bar) and 4 h (dashed bar) against *B. cereus* (on the left) and *P. fluorescens* (on the right). *—Log CFU/cm^2^ reduction was statistical significantly different between time exposures (unpaired *t*-test with Welch’s correction, *p* < 0.05), Table S1: Quantification of total culturable and endospores of *B. cereus* on persister cells after critical biocide treatment, according to described conditions in Table 1. ND—no detectable CFU/cm^2^, Table S2: Formation of persister cells on *P. fluorescens* biofilms—quantification of total cells and viable cells (cells/cm^2^), after GA and PAA treatment. Untreated cells (control samples) corresponded to replacing biocidal solution by sterilized DW. Values are mean ± SDs of three independent assays with two replicates.
