# Supplementary material for: Antimicrobial Susceptibility of Persister Biofilm Cells of Bacillus cereus and Pseudomonas fluorescens
Source: Microorganisms. 2022 Jan 13;10(1):160. doi: 10.3390/microorganisms10010160 (PMC8779418; doi:10.3390/microorganisms10010160)
Supplement: Supplementary file 1 [file microorganisms-10-00160-s001.zip › microorganisms-1532697-supplementary.pdf]

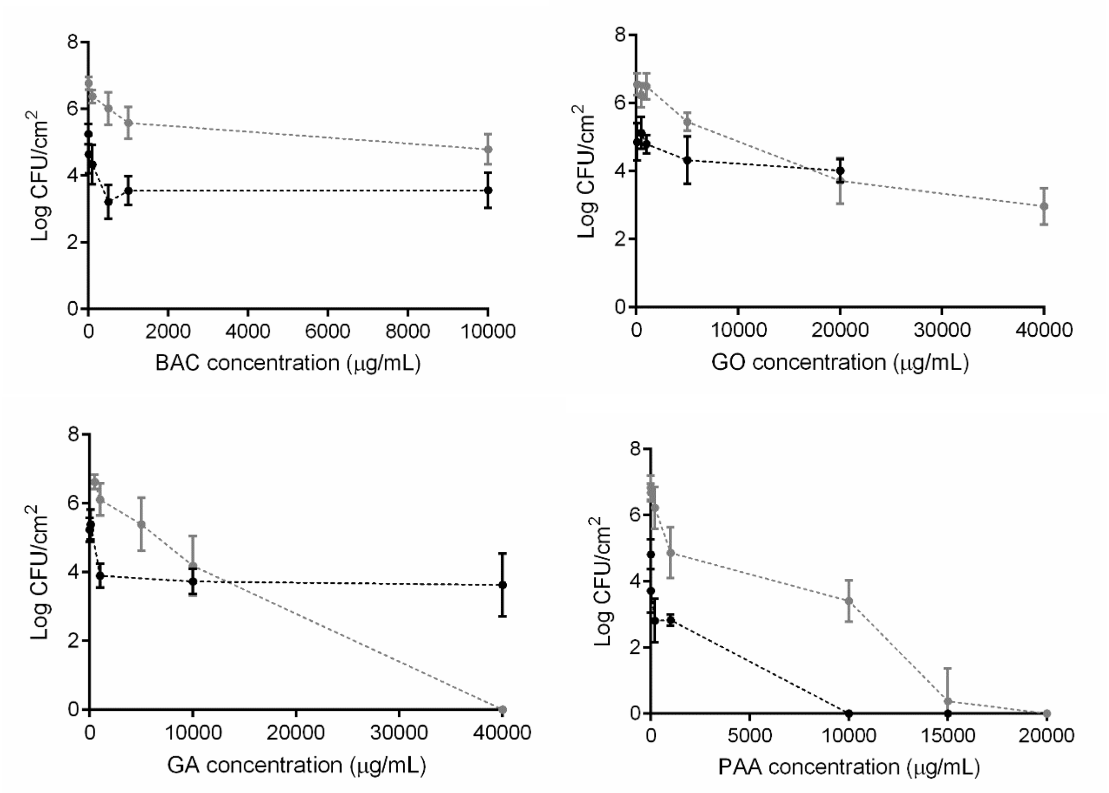


**Figure S1.** Log CFU/cm$^2$ of 48 h-old *B. cereus* (in black) and *P. fluorescens* biofilms (in grey) treated with selected biocides (BAC, GA, GO and PAA) at different concentrations (in µg/mL) for 30 min. Values are means ± SDs of three independent experiments.

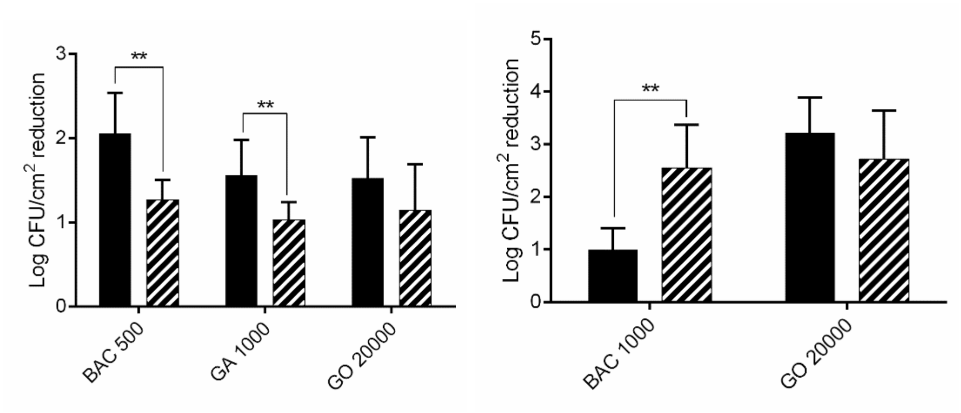


**Figure S2**. Antimicrobial activity of BAC, GA and GO at high concentrations (in µg/mL) for 30 min (full bar) and 4 h (dashed bar), against *B. cereus* (on the left) and *P. fluorescens* (on the right). * – log CFU/cm$^2$ reduction was statistical significantly different between time exposures (unpaired *t*-test with Welch's correction, $P < 0.05$).

**Table S1.** Quantification of total culturable and endospores cells of *B. cereus* on persister cells after critical biocide treatment, according to described conditions in Table 1. ND – no detectable CFU/$cm^2$.

| | **Total (log CFU/$cm^2$)** | **Endospores (log CFU/$cm^2$)** |
|---|---|---|
| **BAC** | 3.2±0.5 | 2.7±0.6 |
| **GA** | 3.9±0.3 | 3.2±0.7 |
| **GO** | 4.0±0.3 | 3.3±0.6 |
| **PAA** | ND | ND |
| **Untreated cells (control)** | 5.3±0.5 | 3.5±0.5 |

**Table S2.** Formation of persister cells on *P. fluorescens* biofilms – quantification of total cells and viable cells (cells/$cm^2$), after GA and PAA treatment. Untreated cells (control samples) corresponded to replacing biocidal solution by sterilized distilled water. Values are mean ± SDs of three independent assays with two replicates.

| | **Total cells (log cells/$cm^2$)** | **Viable cells (log CFU/$cm^2$)** |
|---|---|---|
| **GA** | 6.6±0.1 | 4.8±0.7 |
| **PAA** | 6.7±0.2 | 4.7±0.7 |
| **Untreated cells (control)** | 6.2±0.2 | 6.0±0.2 |
